# Interfacial Gap Prediction in Laser Welding of Pure Copper Overlap Joints Using Multiple Sensors

**DOI:** 10.3390/ma18225189

**Published:** 2025-11-14

**Authors:** Hyeonhee Kim, Cheolhee Kim, Minjung Kang

**Affiliations:** 1 Flexible Manufacturing R&D Department, Korea Institute of Industrial Technology, Incheon 21999, Republic of Korea; 2080khh@gmail.com; 2 School of Mechanical Engineering, Yonsei University, Seoul 03722, Republic of Korea; 3 Department of Mechanical and Materials Engineering, Portland State University, Portland, OR 97229, USA

**Keywords:** copper overlap welding, laser welding, Interfacial gap, deep learning, multi-sensor

## Abstract

In this study, a novel approach was proposed for predicting the interfacial gap in copper overlap joints by using deep learning and multi-sensor fusion. In this method, an image sensor, a spectrometer, and optical sensors tomography (OCT) sensors were used to develop and validate deep learning models under various gap conditions. The results revealed that the variation in melt pool dimensions, changes in keyhole behavior, intensity variations at specific wavelengths, and keyhole depth derived from the OCT data could be used to accurately predict the interfacial gap. Among the proposed models, a binary gap classification model achieved the highest accuracy of 98.8%. The spectrometer was the most effective sensor in this study, whereas the image and OCT sensors provided complementary data. The best performance was achieved by fusing all three sensors, which emphasizes the importance of sensor fusion for precise gap prediction. This study provides valuable insights into improving weld quality assessment and optimizing manufacturing processes.

## 1. Introduction

Optical coherence tomography (OCT) is increasingly recognized as a versatile technique for inline quality monitoring throughout different welding stages—pre-, in-, and post-process. In the pre-process stage, it enables efficient tracking of joint seam alignment [1]. During welding, OCT provides real-time monitoring of critical parameters such as melt pool dynamics [2] and keyhole depth [3,4,5]. In the post-process stage, it supports inspection of the solidified weld geometry [6] to ensure overall weld quality. Moreover, by scanning the OCT laser beam relative to the processing laser beam, OCT enables simultaneous measurement of keyhole depth and melt pool geometry, delivering highly precise in situ monitoring coaxial with the process laser beam [7].

OCT has been applied to enable in situ monitoring and closed-loop control of the laser welding process [5,8,9,10]. It has been successfully employed to measure weld penetration depth and keyhole geometry in real time with high spatial resolution. Blecher et al. [3] monitored keyhole depth using OCT and validated the accuracy of the measured data by comparing it with longitudinal metallographic cross-sections of various metal alloys. Fetzer et al. [11] correlated keyhole depth data from high-speed X-ray imaging with inline OCT measurements, improving accuracy through noise-reduction filters calibrated against X-ray ground truth. Kogel-Hollacher et al. [12] introduced a short-coherence interferometry-based technology for in situ keyhole depth measurements during laser processing, achieving deviations within ±10 µm under typical conditions. He et al. [13] developed a real-time laser welding depth inspection system based on spectral-domain OCT combined with HDBSCAN clustering, achieving an average depth measurement error below 5%. Schmoeller et al. [4,5] enhanced inline weld depth prediction and control by applying an intelligent algorithm to OCT signals within a keyhole, reducing the mean deviation between estimated and actual weld depths from 30% [4] to 1.7% [5]. Will et al. [2] employed an OCT sensor in laser welding at laser powers between 3000 W and 6000 W and welding speeds ranging from 6 m/min to 80 m/min, classifying weld stability based on measured surface topography with a resolution of approximately 0.08 mm. Collectively, these studies demonstrate that OCT provides significant advantages in terms of depth resolution, real-time feedback, and adaptability to different materials and process dynamics. These findings strongly support OCT as a robust and scalable sensing platform for advanced laser welding applications.

Despite its advantages, OCT faces limitations under the complex and dynamic conditions of laser welding. Its reliability declines particularly with highly reflective materials such as aluminum and copper, where beam scattering and weak signal return hinder accurate measurement. To address this, machine learning (ML) models have been increasingly adopted for real-time classification and regression tasks, as they can learn the discrepancy between predicted and actual values. For improved accuracy and robustness, ML models are often integrated with multi-sensor configurations, combining OCT with acoustic signals [14,15], thermo-cameras [16,17], image sensors [18], spectrometers [19,20], and photodiodes [21,22,23].

To address the limitations of OCT, recent studies have increasingly explored sensor fusion approaches supported by machine learning. Brezan et al. [22] demonstrated that integrating photodiodes with OCT achieved 87% accuracy in weld quality classification. Cao et al. [15] proposed a monitoring method for aluminum laser welding that combined acoustic and photodiode signals with convolutional neural network (CNN) algorithms, achieving 94.34% accuracy. Cai et al. [24] employed a high-speed camera with an image-fusion method based on two-dimensional CNN models to recognize the penetration state with an accuracy of up to 99.86%. Kim et al. [25] demonstrated the fusion of an image sensor and a spectrometer to estimate penetration depth during laser welding of DP780 steel, achieving a mean absolute error of 0.049 mm.

Copper welding, particularly of thin foils, has become increasingly important in electric vehicle battery modules, power electronics, and thermal management systems, where reliable joining is essential for both electrical and mechanical performance [26]. These joints are typically formed as non-visible overlap configurations (e.g., busbar-to-tab, tab-to-tab, or tab-to-collector plate). However, deformation of materials and tolerances in the jigging system can lead to the formation of interfacial gaps. Even a small gap can cause insufficient fusion, pore formation, or reduced joint strength, highlighting the necessity of in situ gap monitoring.

In this study, we propose a deep learning-based approach to predict interfacial gaps during overlap laser welding of copper sheets using a multi-sensor fusion framework. To overcome the inherent limitations of individual sensors, our sensing architecture integrates image, spectral, and geometric data obtained from an image sensor, a spectrometer, and an OCT sensor. The analysis focuses on the relative contribution of each modality to identify the most informative features for interfacial gap detection. Based on these insights, we establish a data-driven framework to enable intelligent, scalable weld quality monitoring in copper laser welding.

## 2. Experimental Setup and Data Preparation

### 2.1. Experimental Setup

**Figure 1:**
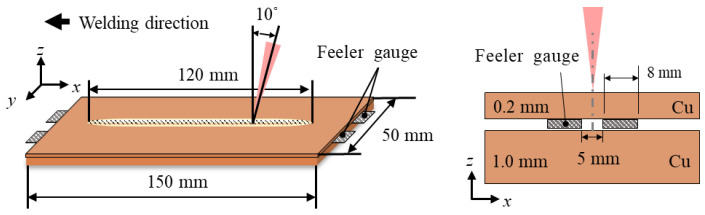
Experimental setup.

The base materials used in the experiments were C1100 copper (thickness: 0.2 mm, Cu 99.98%) and C1020 copper (thickness: 1.0 mm, Cu 99.959%). All specimens were machined to dimensions of 50 mm × 150 mm (Figure 1). Laser welding was performed in an overlap joint configuration, with the C1100 sheet placed on top of the C1020 sheet.

To introduce interfacial gap conditions, feeler gauges with thicknesses ranging from 0.02 to 0.1 mm were inserted between the upper and lower sheets. These gauges were positioned symmetrically on either side of the weld line, 2.5 mm from the centerline, and extended 8 mm along the welding direction (Figure 1).

The laser beam was generated by a fiber laser (YLS-6000, IPG Photonics, Oxford, MA, USA) and delivered through a 200 μm optical fiber to a focusing optic (D30, IPG Photonics, Oxford, MA, USA) with a focal length of 200 mm. At the focal position, the measured beam diameter was 270 μm. Welding was conducted at laser powers of 2.0 kW, 2.25 kW, and 2.5 kW, while maintaining a constant travel speed of 9 m/min (Table 1). Two weldments were produced for each experimental condition. The laser beam was applied with a 10° push angle, and no shielding gas was used during welding (Figure 1).

**Figure 2:**
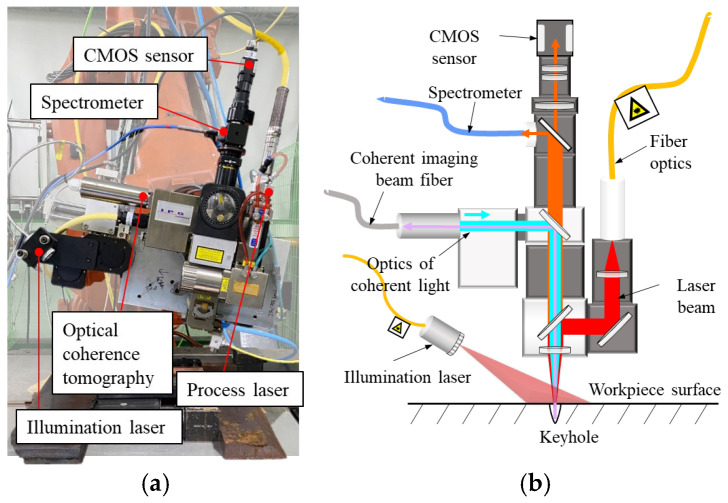
(**a**) Age of equipment and (**b**) schematic diagram of coaxial process monitoring devices.

To extract features related to the interfacial gap, three sensors—a CMOS camera (UI-6140CP-M-GL.Rev.2, IDS, Obersulm, Germany), a spectrometer (HR-4000, Ocean Optics, Dunedin, FL, USA), and an OCT sensor (LDD-700, IPG Photonics, Oxford, MA, USA)—were coaxially mounted on the focusing optic (Figure 2). The sensors were synchronized via a trigger system, allowing simultaneous acquisition of all data streams.

CMOS camera images were captured at a resolution of 202 × 472 pixels and a frame rate of 500 Hz, with an integration time of 0.829 ms. The images were filtered using a band-pass filter with a full width at half maximum (FWHM) of 10 nm and a maximum transmission loss of 15%. A 980 nm diode laser with an output power of 55 W was used as the illumination source to enhance image visibility near the molten pool. The illumination beam was projected onto the welds from the front side of the welding direction, forming an angle of approximately 60° with the process laser. Due to the incidence angle and working distance, the illuminated region on the specimen exhibited an elliptical shape, with minor and major axes of approximately 17 mm and 30 mm, respectively.

The spectrometer measured light intensity over a wavelength range of 194–1127 nm, with a sampling rate of 100 Hz, an optical resolution of 0.47 nm, and an integration time of 10 ms. The OCT system, based on the Michelson interferometer principle and operating in the 800–900 nm wavelength range, enabled measurements of keyhole depth, bead height, and bead width. The system specifications include an axial (vertical) resolution of 20–50 µm over a 6 mm axial field of view, and a lateral resolution of approximately 15 µm with a lateral scanning range of 10 mm. With a maximum sampling frequency of 250 kHz, the OCT system provided high-speed acquisition of geometric feature data.

### 2.2. OCT Performance Evaluation

**Figure 3:**
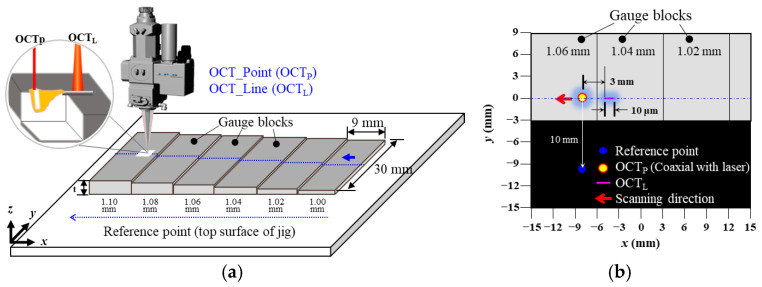
Schematic of OCT evaluation setup using gauge blocks, illustrating the relative OCT beam position OCT_P_ and OCT_L_: (**a**) skewed 3D view and (**b**) top view.

To detect deviations in gap thickness, the accuracy of the OCT sensor must be finer than the thickness of the foil used. Since the resolution of the OCT sensor is highly dependent on the optical system, the accuracy and limitations of the OCT sensor were evaluated prior to the laser welding trials. To verify the resolution of the OCT system, gauge blocks were prepared with thickness intervals of 0.02 mm, ranging from 1.00 to 1.10 mm (1.00, 1.02, 1.04, 1.06, 1.08, and 1.10 mm). These gauge blocks, which have dimensions of 30 mm in width and 9 mm in length, were sequentially arranged as illustrated in Figure 3. Surface height measurements were conducted at travel speeds of 1 and 5 m/min using a linear motion stage, and each test was repeated three times to ensure measurement reliability. As depicted in Figure 3, the OCT sensor was utilized in tow modes depending on the measurement purpose: point measurement for detecting keyhole depth (OCT_P_) and line measurement (OCT_L_) for evaluating average bead height. The OCT_L_ measurement was performed 3 mm away from the OCT_P_ location, with a sampling interval of 10 μm. A reference point was located 10 mm away from the OCTP location. Since OCTP detects the keyhole depth located below the reference surface, its values are recorded as negative, whereas OCTL measured the bead height above the reference surface, resulting in positive values. The raw OCT data were processed using a moving-average smoothing method with a window size of 500 µm to reduce signal noise and extract geometric features. The OCT signal, originally sampled at 135 kHz, was downsampled to 500 Hz by averaging every 270 consecutive data points.

**Figure 4:**
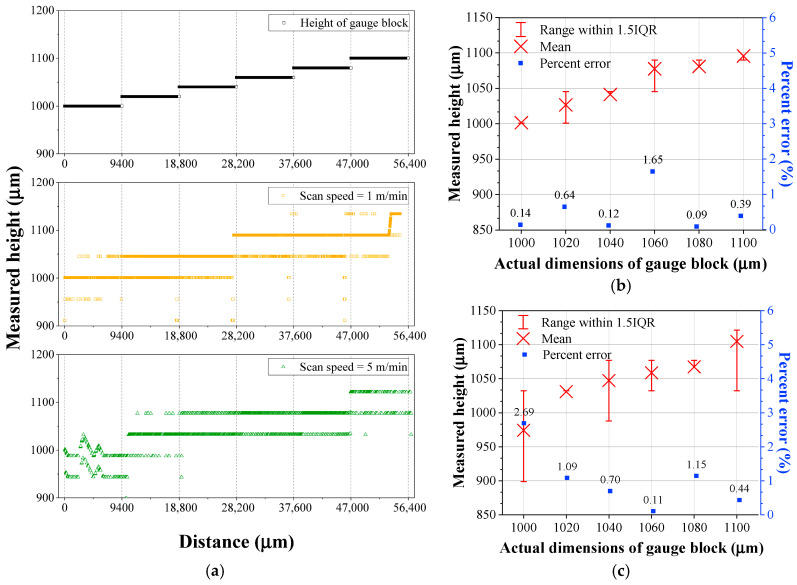
(**a**) Height profile measured by OCT sensor for gauge blocks with varying heights, and plots of height deviations and errors at different speeds: (**b**) 1 m/min and (**c**) 5 m/min.

The as-measured scanning height of the gauge block was presented in Figure 4. Figure 4a displayed the raw data obtained using the OCT system, revealing that the OCT sensor did not accurately detect the surfaces of the gauge blocks. Although the actual specimen heights consisted of six distinct levels, the measured heights did not show distinct separation. In Figure 4a, it should be noted that the very low height measurements at the block interfaces originated from the slight gap between the gauge blocks. Figure 4b,c summarized the measured heights at travel speeds of 1 m/min and 5 m/min, respectively. At 1 m/min, percent errors of less than 1% were observed in all conditions except for the 1.06 mm condition, while at 5 m/min, errors exceeding 1% occurred in half of the conditions (three out of six). As the travel speed increased, the average percent error rose from 0.51% to 1.03%.

**Figure 5:**
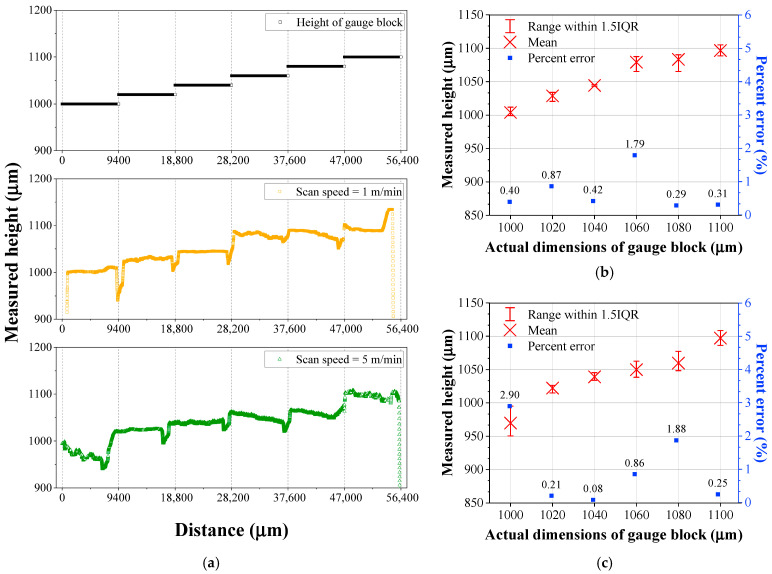
(**a**) Moving-averaged height profile for gauge blocks with varying heights, and plots of height deviations and errors at different speeds: (**b**) 1 m/min and (**c**) 5 m/min.

Figure 5 shows the modified height data obtained by applying a moving-average smoothing with a window size of 500 μm to the raw OCT sensing data. After moving-average smoothing, the height of the gauge blocks was detected more accurately, and the percent error decreased compared to the non-smoothed data shown in Figure 4. Although the percent error still increased as increase in travel speed, the shape of the gauge blocks was detected more reliably. Additionally, the deviation was significantly reduced, and the number of errors exceeding 1% decreased. However, the average percent error tended to increase after smoothing. Specifically, the percent errors after smoothing were 0.68% at 1 m/min and 1.02% at 5 m/min.

The OCT sensor was well-suited for measuring geometrical features such as the keyhole; however, its resolution was constrained by the detection methodology and optical system. The optical sensor installed in the OCT system has a pixel size of 45 μm in both horizontal and vertical directions, which limited its ability to detect fine gaps smaller than 0.04 mm. As a result, the OCT system alone was insufficient to resolve the 20 μm height differences in the gauge blocks and the gap variations introduced during the experiment. These limitations highlight the need for improved sensing strategies.

### 2.3. Data Preparation

#### 2.3.1. Multi-Sensor Data Acquisition

**Figure 6:**
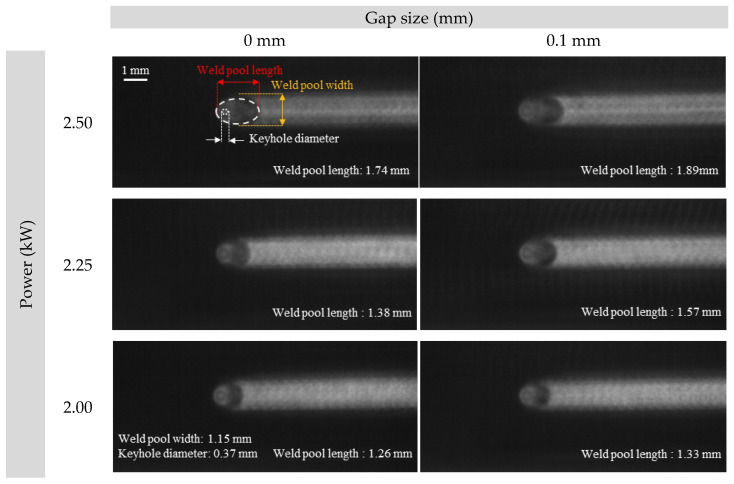
Re-sampled CMOS images according to gap size and laser power.

The CMOS image sensor was installed coaxially to observe the keyhole, molten pool, and weld bead. The CMOS images were resampled to minimize the effect of weld pool fluctuation by averaging every 100 images. As shown in Figure 6, a longer molten pool was observed when a gap existed at the interface compared to the condition without a gap. The difference in molten pool length between gap and no-gap condition increased with laser power. As the power varied from 2.0 to 2.5 kW, the deviation increased from 0.0 mm to 0.19 mm. However, the width of molten pool and the keyhole diameter remained constant at 1.15 mm and 0.37 mm, regardless of the gap or laser power.

**Figure 7:**
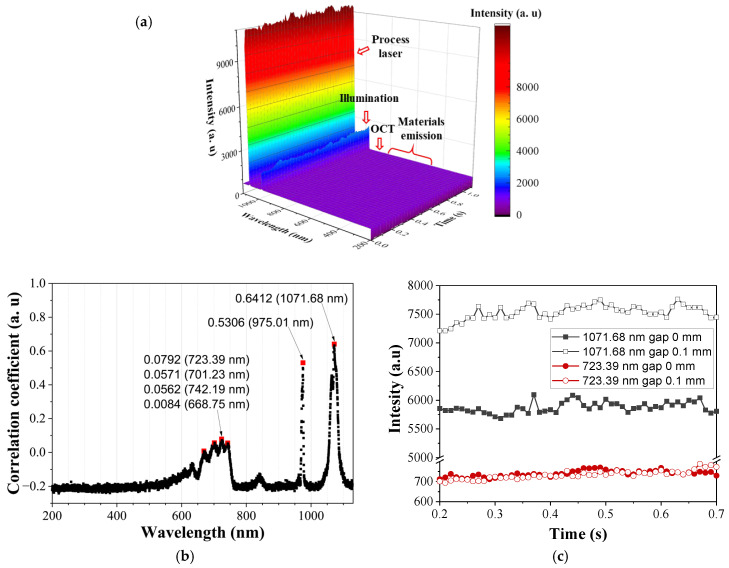
(**a**) Spectral signal profiles, (**b**) correlation coefficient related to the gap, and (**c**) spectral intensity at specific wavelengths (laser power = 2.5 kW and welding speed = 9 m/min).

The spectral intensity corresponding to material emission (visible range), process laser beam reflection (around 1070 nm), illumination laser reflection (around 980 nm), and OCT laser reflection (around 980 nm) was detected, as shown in Figure 7a. Both wavelength and intensity changed significantly depending on the interfacial gap size, showing strong correlations at specific wavelengths. In the visible range, the copper spectral peak at 723.39 nm had a correlation coefficient of 0.079, whereas in the IR range, the process laser wavelength at 1071.68 nm exhibited a much higher correlation coefficient of 0.6412 (Figure 7b). The spectral intensity at 1071.68 nm varied markedly with the gap condition, while that at 723.39 nm remained almost constant.

**Figure 8:**
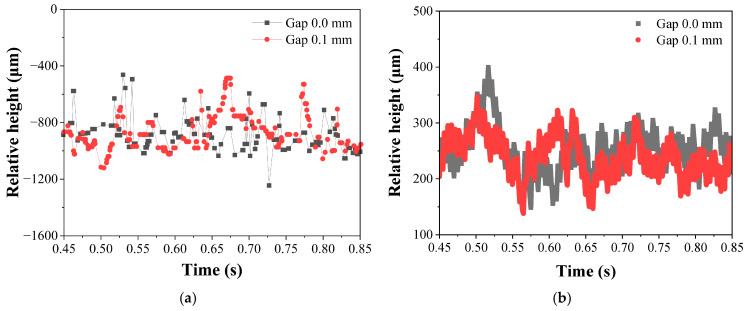
Geometrical feature measured by (**a**) OCT_P_ and (**b**) OCT_L_ depending on the gap (laser power = 2.5 kW and welding speed = 9 m/min).

The OCT sensing data for keyhole depth and bead height measurements were presumed to be suitable due to the negligible differences related to the size of the interfacial gap, as shown in Figure 8. Correlation analysis revealed that the bead height exhibited a correlation coefficient of −0.276, whereas the keyhole depth showed a coefficient of 0.068, indicating that the relationship between bead height and gap was statistically more significant.

#### 2.3.2. Input Data Pre-Processing

**Figure 9:**
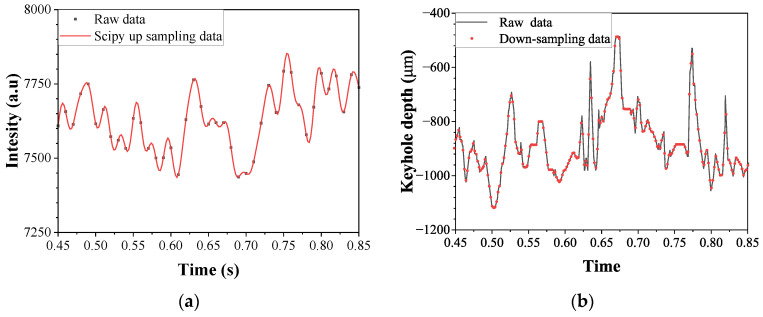
Time-series signal for (**a**) spectrometer at 1071.68 nm and (**b**) OCT sensor (Laser welding parameter: laser power = 2.5 kW, welding speed = 9 m/min, gap = 0.1 mm)).

For the development of the prediction model, data collected from each sensor were normalized in terms of sampling frequency. Coaxial images were acquired at 500 Hz and used in their raw form. Spectrometer signals, consisting of 3648 wavelength channels, were collected at 100 Hz and upsampled to 500 Hz using a Fourier-based interpolation method [27]. OCT signals, originally sampled at 135 kHz, were downsampled to 500 Hz by applying averaging to the time-series data. As shown in Figure 9a,b, the transformed spectrometer and OCT data preserved the key characteristics of the original signals. The final input for the time-series prediction model consisted of a 472 × 202 pixel coaxial image, 3648 spectrometer features, and a single OCT signal. Python v3.8.8 was used for data processing, and all codes were developed and executed in Jupyter Notebook v6.0.0.

#### 2.3.3. Output Data Labeling

**Figure 10:**
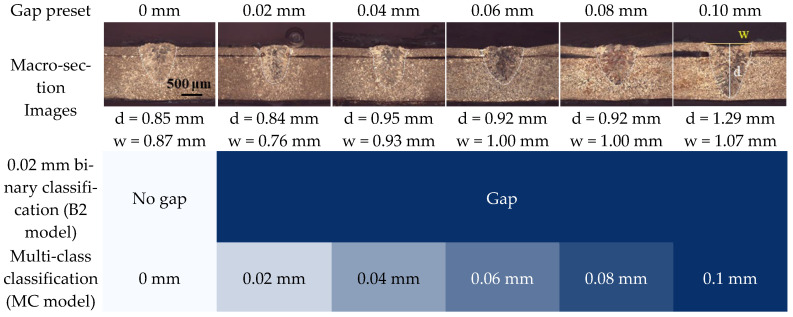
Definition of output classes.

The interfacial gap during welding was precisely configured using feeler gauges. To verify the actual gap after welding, specimens were randomly sectioned (Figure 10) and the measured gap heights were compared to the nominal filler gauge values. Across three measured coupons per gap, the average deviation between the set and measured gaps was 6.67 μm.

Two classification models were developed to predict the interfacial gap: a binary classification model (B2 model) and a multi-class classification model (MC model). In the B2 model, the threshold was defined as 0.02 mm. Gaps of 0 mm were labeled as the “no gap” class, while all other cases were labeled as the “gap” class. In the MC model, six discrete classes were defined according to the preset gap sizes (Figure 10).

## 3. Deep Learning Model Structure

**Figure 11:**
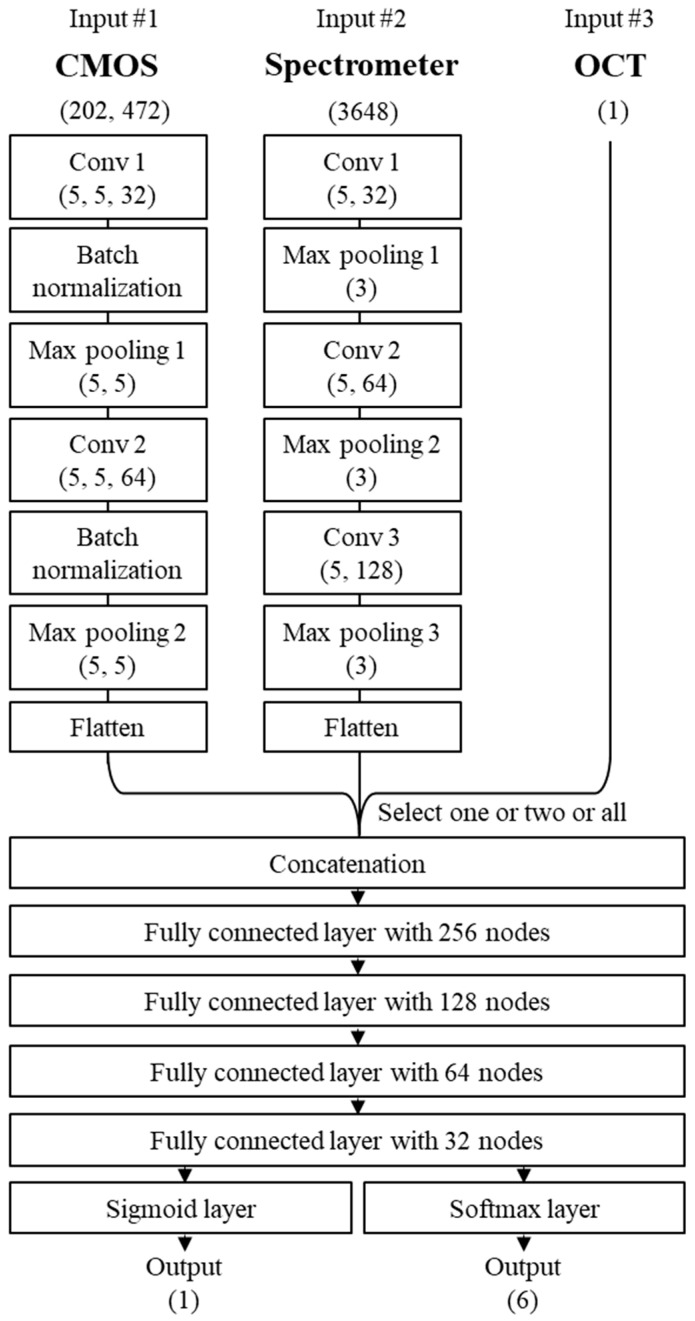
Architecture of the developed CNN structure for interfacial gap prediction.

A B2 and a MC model were developed to predict the interfacial gap. Both models, shown in Figure 11, used a 2D-CNN for image data, which was composed of two blocks of convolution, batch normalization, and max pooling layers. For the spectrometer data, a 1D-CNN architecture was used, consisting of three similar blocks. The OCT data was incorporated into the network as a scalar input. Depending on the OCT data used for training, the models were named the OCT_P_-MC model (using keyhole depth data) and the OCT_L_-MC model (using bead height data).

Each data modality provided complementary information to the network: the 2D-CNN extracted spatial features of the molten pool from coaxial images, while the 1D-CNN processed wavelength-dependent emission profiles from the spectrometer. The scalar OCT input supplied geometric information corresponding to either keyhole depth or bead height. After concatenation, the combined features enabled the model to learn correlations between optical emissions and geometric variations. The B2 model classified the presence or absence of a gap, whereas the multi-class (MC) model predicted six discrete gap intervals.

The outputs from the 2D-CNN (image), the 1D-CNN (spectrometer), and the scalar OCT input were flattened and concatenated. This combined feature vector was then fed into a fully connected network (FCN) with four dense layers. To prevent overfitting, kernel regularizers with parameters of L1 = 0.001 and L2 = 0.001 were applied to two of the dense layers. The rectified linear unit (ReLU) function was used as the activation function for all hidden nodes. For the output nodes, the sigmoid function was used in the B2 model, while the softmax function was used in the MC model.

## 4. Results

### 4.1. Model Training

**Figure 12:**
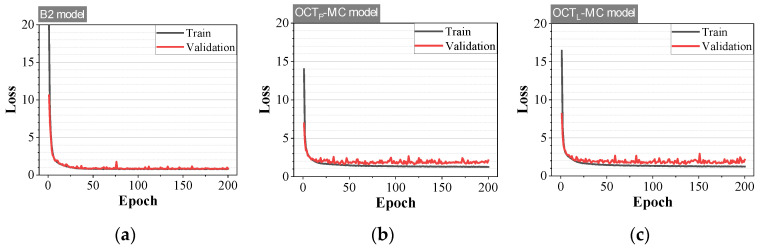
Training and validation losses for CNN models: (**a**) B2, (**b**) OCT_P_-MC, and (**c**) OCT_L_-MC.

A total of 13,680 data points were collected through data pre-processing. The dataset was randomly divided into training, validation, and test sets in proportions of 70% (8820 points), 15% (1890 points), and 15% (1890 points), respectively. For the B2 model, a binary crossentropy function was used as the loss function, whereas a categorical crossentropy function was applied for the MC model [28]. The Adam optimizer was employed with a learning rate of 0.001, β_1_ = 0.9, β_2_ = 0.999, and ε = 10^−8^ [29]. Training was conducted over 200 epochs with a mini-batch size of 32. The training error decreased sharply during the initial epochs and converged after approximately the 25th epoch (Figure 12). No overfitting was observed in any of the models.

**Figure 13:**
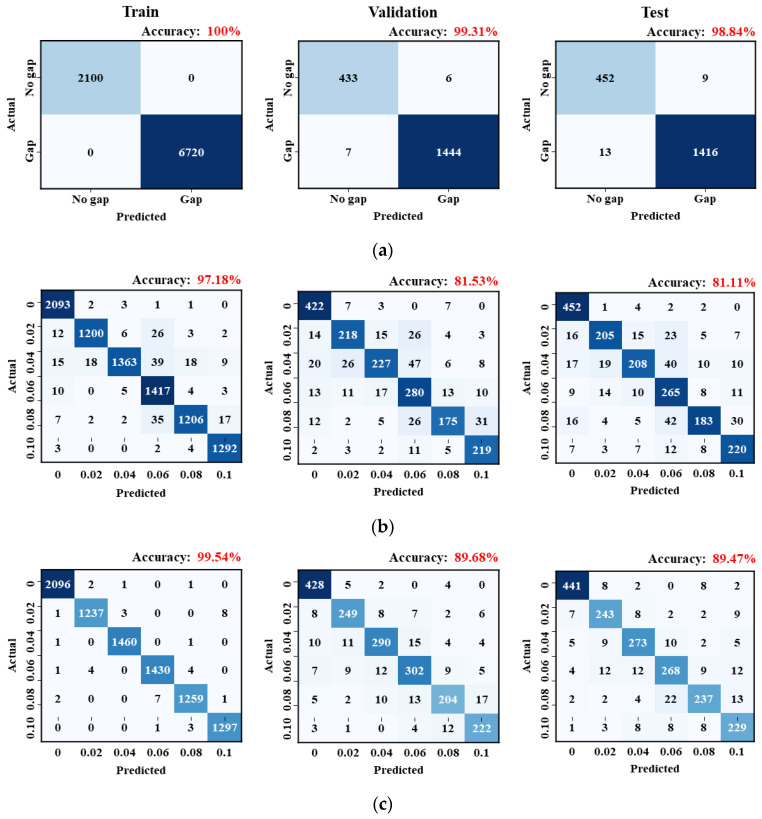
Training results for CNN models: (**a**) B2, (**b**) OCT_P_-MC, and (**c**) OCT_L_-MC.

The accuracy of all trained models exceeded 89%, as shown in Figure 13 and Table 2. Although the training accuracy approached 100% across all three models, the validation accuracy remained at lower levels. For the test dataset, the B2 model achieved the highest performance with an accuracy of 98.84% (Figure 13).

Among the MC classification models, the OCT_P_-MC model exhibited lower accuracy compared to the OCT_L_-MC model. The test accuracy of the OCT_P_-MC model was 81.11%, whereas that of the OCT_L_-MC model reached 89.47%. Evaluation metrics, including precision and recall for the B2 and MC models, are presented in Table 3, Table 4 and Table 5. Precision is defined as the proportion of predicted “Gap” instances that were actually “Gap,” while recall refers to the proportion of true “Gap” instances correctly identified by the model.

This study aimed to determine the criteria for predicting interfacial gap sizes based on the characteristics of each sensor corresponding to various gap sizes. To achieve this, precision and recall, which are performance evaluation metrics derived from the confusion matrix, were compared. The B2 model, which performed binary classification based on the presence or absence of an interfacial gap, achieved the highest precision and recall (Table 3). Among the MC models, the OCT_L_-MC model outperformed the OCT_P_-MC model across all gap sizes (Table 4 and Table 5).

### 4.2. Evaluation of Sensor Performance

**Figure 14:**
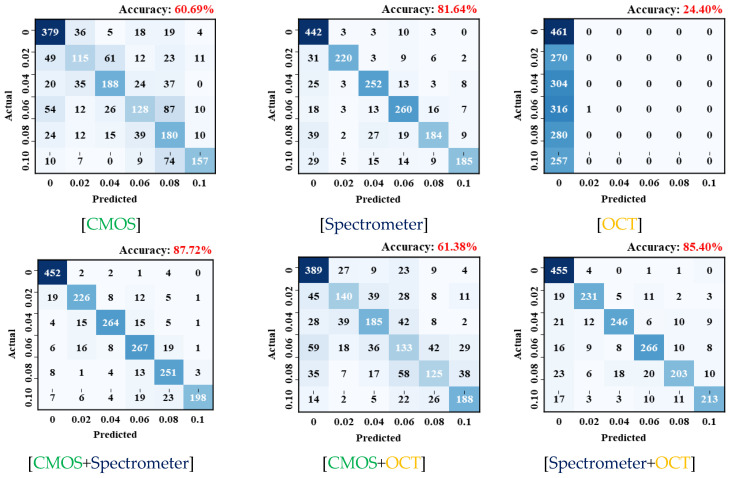
Trained results for sole and dual sensor configurations.

To evaluate the influence of each sensor in the OCT_L_-MC model—which demonstrated the highest accuracy among the MC classification models—training was performed using different sensor combinations (Figure 14 and Table 6). Among the single-sensor cases, the spectrometer alone yielded the highest accuracy (81.64%), whereas the OCT sensor alone performed poorly, with an accuracy of only 24.40%. For two-sensor combinations, CMOS + OCT achieved 61.38% accuracy, while spectrometer + OCT reached 85.40%. These results indicate that adding the OCT sensor to either CMOS or spectrometer improves classification performance compared to using CMOS or spectrometer alone. The CMOS + spectrometer combination produced the best result among all two-sensor configurations, with an accuracy of 87.72%.

## 5. Discussion

Based on the evaluation of each sensor’s performance, the spectrometer alone achieved the highest accuracy among the single-sensor models, recording 81.64%. The CMOS sensor reached 60.69%, while the OCT sensor, when used individually, exhibited a substantially lower accuracy of 24.40%, indicating limited capability in classifying interfacial gaps.

The relatively low accuracy of the CMOS sensor can be attributed to the limited sensitivity of image-based features to gap-induced changes. Although the presence of an interfacial gap slightly elongated the molten pool, the overall variation was minimal. This was primarily due to the high welding speed and the intrinsic properties of copper, including its low IR absorptivity and high thermal conductivity, which restricted molten pool expansion and maintained a nearly constant pool width regardless of the gap. Consequently, the visual features extracted from CMOS images provided insufficient variation to effectively distinguish gap conditions.

The spectrometer, by contrast, delivered the most informative signal among the three sensors, exhibiting strong correlations with gap size at specific wavelength regions. Correlation coefficients exceeded 0.6 in the process laser band (1070 nm) and 0.5 in the illumination laser band (980 nm), whereas the material emission band (600–700 nm) showed a correlation below 0.1. The latter result is likely attributable to the Cu-Cu laser welds employed in this study; previous studies [30] have reported higher correlations in this band when heterogeneous materials were employed. Notably, high correlation coefficients were observed around 975 nm (illumination laser) and 1070 nm (process laser), suggesting that a photodetector equipped with an appropriate narrowband optical filter could provide a simpler and faster means to achieve comparable performance in interfacial gap detection.

When used in a sole sensor, the OCT sensor failed to classify gaps effectively, as neither point nor line data were sufficient. However, in combination with other sensors, OCT_L_ provided a more informative feature than OCT_P_. This outcome can be explained by the physical phenomenon wherein the molten pool sags downward due to the presence of an interfacial gap, leading to a reduction in bead height. As shown in Figure 10, bead height decreases in the presence of a gap. Under normal laser welding conditions, bead height remains positive, but in the case of a 0.1 mm gap, the molten pool sagged into the gap, resulting in negative bead height.

## 6. Conclusions

This study proposed a deep learning-based approach for predicting interfacial gaps during overlap joint laser welding of Cu–Cu materials by leveraging a multi-sensor fusion framework. To address the inherent limitations of individual sensors, a high-dimensional data modeling strategy was adopted by integrating image signals (CMOS), spectral signals (spectrometer), and scalar signals (OCT). A total of 13,680 datasets were collected, and both binary (B2) and multi-class (MC) classification models were trained to evaluate the contribution of each sensor and the effectiveness of various sensor combinations. The following conclusions were drawn:

(i) The B2 classification model outperformed other models, achieving the highest test accuracy of 98.84%, indicating strong potential for reliable interfacial gap detection in Cu–Cu welding. Among the MC models, the OCT_L_-MC model, which used bead height instead of keyhole depth, showed superior performance (89.47%).
(ii) The spectrometer was identified as the most influential sensor, achieving the highest standalone accuracy (84.64%) and exhibiting a strong correlation with gap size in specific wavelength bands (correlation coefficient of 0.6412 at 1070 nm). In contrast, the CMOS sensor exhibited relatively lower sensitivity to gap size, owing to the minimal variation in molten pool size under high-speed copper welding.
(iii) The OCT sensor alone was insufficient for effective classification, but when combined with other sensors—particularly through the OCT_L_ approach—it significantly improved model performance. OCT_L_ exhibited a stronger correlation with gap size than OCT_P_, reflecting the physical behavior of the molten pool sagging into the interfacial gap.
(iv) Key features for interfacial gap prediction included molten pool length (from CMOS), spectral intensity variations (from the spectrometer), and bead height changes (from OCT). The deep learning model effectively extracted these features from raw sensor signals, enabling accurate classification based on Cu–Cu welding behavior.

In summary, this study demonstrates that sensor fusion and data-driven modeling are highly effective for detecting interfacial gaps in overlap joint Cu-Cu laser welds. The proposed framework provides a foundation for real-time weld quality monitoring and can be extended to applications such as dissimilar metal welding or adaptive process control.

## Figures and Tables

**Table 1:** Welding variables used in experiment.

Variables	Parameter (Level)
Laser power (kW)	2.5, 2.25, 2.0 (3)
Welding speed (m/min)	9 (1)
Gap (mm)	0, 0.02, 0.04, 0.06, 0.08, 0.1 (6)

**Table 2:** Accuracy of trained models.

Accuracy (%)	B2 Model	OCT_P_-MC	OCT_L_-MC
Training	100	97.18	95.54
Validation	99.31	81.53	89.68
Test	98.84	81.11	89.47

**Table 3:** Evaluation metrics (precision, recall, and F1 score) of B2 model.

	Precision	Recall	F1 Score
0.02 mm binary classification	0.9720	0.9805	0.9760

**Table 4:** Evaluation metrics (precision, recall, and F1 score) of OCT_P_-MC model.

	0 mm	0.02 mm	0.04 mm	0.06 mm	0.08 mm	0.1 mm
Precision	0.874275	0.833333	0.835341	0.690104	0.847222	0.791367
Recall	0.980477	0.756458	0.684211	0.835962	0.653571	0.856031
F1 score	0.924335	0.793037	0.75226	0.756063	0.737903	0.82243

**Table 5:** Evaluation metrics (precision, recall, and F1 score) of OCT_L_-MC model.

	0 mm	0.02 mm	0.04 mm	0.06 mm	0.08 mm	0.1 mm
Precision	0.958696	0.877256	0.889251	0.864516	0.890977	0.848148
Recall	0.956616	0.896679	0.898026	0.845426	0.846429	0.891051
F1 score	0.957655	0.886861	0.893617	0.854864	0.868132	0.86907

**Table 6:** Classification accuracy for sole and dual sensor configurations.

	CMOS	Spectrometer	OCT	CMOS + Spectrometer	CMOS + OCT	Spectrometer + OCT
Accuracy (%)	60.69	81.64	24.40	87.72	61.38	85.40

## Data Availability

The original contributions presented in this study are included in the article. Further inquiries can be directed to the corresponding author.
